# Frequency of health‐care utilization by adults who use illicit drugs: a systematic review and meta‐analysis

**DOI:** 10.1111/add.14892

**Published:** 2020-02-10

**Authors:** Dan Lewer, Joseph Freer, Emma King, Sarah Larney, Louisa Degenhardt, Emily J. Tweed, Vivian D. Hope, Magdalena Harris, Tim Millar, Andrew Hayward, Dan Ciccarone, Katherine I. Morley

**Affiliations:** ^1^ UCL Collaborative Centre for Inclusion Health Institute of Epidemiology and Health Care UCL London UK; ^2^ National Addictions Centre, Institute of Psychiatry, Psychology and Neuroscience, King's College London London UK; ^3^ Centre for Primary Care and Public Health Queen Mary University of London London UK; ^4^ National Drug and Alcohol Research Centre (NDARC) University of New South Wales Randwick, NSW Australia; ^5^ MRC/CSO Social and Public Health Sciences Unit University of Glasgow Glasgow UK; ^6^ Public Health Institute Liverpool John Moores University Liverpool UK; ^7^ Department of Public Health, Environments and Society London School of Hygiene and Tropical Medicine , London UK; ^8^ Centre for Mental Health and Safety The University of Manchester Manchester UK; ^9^ Department of Family and Community Medicine University of California San Francisco CA USA; ^10^ Centre for Epidemiology and Biostatistics, Melbourne School of Global and Population Health The University of Melbourne Melbourne Australia

**Keywords:** Health services, Hospitals, Opiates, Primary Health Care, Stimulants, Substance‐Related Disorders

## Abstract

**Aims:**

To summarize evidence on the frequency and predictors of health‐care utilization among people who use illicit drugs.

**Design:**

Systematic search of MEDLINE, EMBASE and PsychINFO for observational studies reporting health‐care utilization published between 1 January 2000 and 3 December 2018. We conducted narrative synthesis and meta‐analysis following a registered protocol (identifier: CRD42017076525).

**Setting and participants:**

People who use heroin, powder cocaine, crack cocaine, methamphetamine, amphetamine, ecstasy/3,4‐methyl​enedioxy​methamphetamine (MDMA), cannabis, hallucinogens or novel psychoactive substances; have a diagnosis of ‘substance use disorder’; or use drug treatment services.

**Measurements:**

Primary outcomes were the cumulative incidence (risk) and rate of care episodes in three settings: primary care, hospital admissions (in‐patient) and emergency department (ED).

**Findings:**

Ninety‐two studies were included, 84% from North America and Australia. Most studies focused on people using heroin, methamphetamine or crack cocaine, or who had a diagnosis of drug dependence. We were able to conduct a meta‐analysis of rates across 25 studies reporting ED episodes and 25 reporting hospital admissions, finding pooled rates of 151 [95% confidence interval (CI) = 114–201] and 41 (95% CI = 30–57) per 100 person‐years, respectively; on average 4.8 and 7.1 times more often than the general population. Heterogeneity was very high and was not explained by drugs used, country of study, recruitment setting or demographic characteristics. Predictors of health‐care utilization were consistent across studies and included unstable housing, drug injection and mental health problems. Opioid substitution therapy was consistently associated with reduced ED presentation and hospital admission. There was minimal research on health‐care utilization by people using ecstasy/MDMA, powder cocaine, hallucinogens or novel psychoactive substances.

**Conclusions:**

People who use illicit drugs are admitted to emergency department or hospital several times more often than the general population.

## Introduction

The use of illicit drugs is associated with health, social and economic problems. People who are dependent on illicit drugs generally have poor health outcomes, with cohort studies finding mortality rates of up to 15 times the general population, although this varies widely by population and setting 1, 2. As well as overdose, there is excess risk of cancers, cardiovascular, respiratory and liver diseases 3, 4, 5. Excess disease may be due to both the direct effects of illicit drugs and accompanying life circumstances. For instance, people who use illicit drugs are vulnerable to homelessness, imprisonment and other forms of social exclusion 6, and have high rates of tobacco smoking and harmful alcohol consumption. There are diverse subgroups of people who use drugs, and people who smoke cannabis or use illicit drugs occasionally may have better health outcomes than people who use drugs such as heroin, crack cocaine and methamphetamine 7, 8.

Despite the high need for health care, qualitative research has identified multiple barriers for people who use illicit drugs. Health professionals may have negative perceptions of patients who use illicit drugs, including poor motivation, seeking prescriptions for non‐medical purposes and violent behaviour, and may feel they lack training and skills to address the needs of this group 9. Patients report that staff have stigmatizing attitudes and that there are barriers to attending appointments, such as transport costs and inflexible time‐slots 10. People who use drugs may delay treatment due to normalization of pain, fear of stigma in services and concern about inadequate opioid substitution and pain control when admitted to hospital 11. These barriers mean that symptoms may not be addressed, leading to presentation late in the course of a disease and use of emergency care. People who use illicit drugs face distinct challenges to health‐care access related to criminalization and social exclusion. We have therefore chosen to focus on this group, rather than include people who use alcohol, tobacco or other legal drugs.

Studies of patients visiting emergency departments (ED) have found that 10–20% report recent use of illicit drugs 12, 13, 14, much higher than the general population, and diagnoses of drug dependence are common among frequent ED users 15, 16. Frequent ED users are particularly likely to use drugs 17. Such observations have led to a perception that people who use drugs are reliant upon ED services, but there is limited population‐based research into the frequency and patterns of health‐care utilization in this group. We aimed to (1) describe the frequencies of health‐care utilization reported in observational studies of people who use illicit drugs and calculate pooled averages; (2) compare the frequency of health‐care utilization to the general population; and (3) summarize evidence on the predictors and causes of health‐care utilization.

## Methods

### Review protocol

We conducted a systematic review following the Preferred Reporting Items for Systematic Review and Meta‐Analysis (PRISMA) guidelines 18. A protocol for this review has been registered with PROSPERO (identifier: CRD42017076525).

### Search strategy

We searched Medline, PsychINFO and EMBASE from 1 January 2000 to 27 September 2017 using keywords and MeSH terms related to substance use, health‐care utilization and observational study designs (full terms included in the Supporting information). We also included studies from a manual search of references. On 3 December 2018 we updated our search, using the same databases, search terms and inclusion criteria.

### Study inclusion and exclusion criteria

We included English‐language cohort and cross‐sectional studies where 75% or more of participants had recently used illicit drugs. Illicit drugs were defined as heroin, powder cocaine, crack cocaine, methamphetamine, amphetamine, ecstasy/4‐methyl​enedioxy​methamphetamine (MDMA), cannabis, hallucinogens or novel psychoactive substances. We also included individuals who had had a diagnosis of ‘substance use disorder’ or were recruited from drug treatment services, where we were able to determine that at least 75% used illicit drugs rather than alcohol only. Primary outcomes were the rate or cumulative incidence of ED episodes, hospital admissions and primary care presentation. We excluded studies of participants recruited from acute health‐care services (such as ED), who had acute disease (such as hepatitis A), who were pregnant or were aged less than 18 years. We also excluded studies with fewer than 30 participants or less than 30 days of observation per participant.

### Study quality assessment

Methodological quality was assessed using a modified Newcastle‐Ottowa scale 19 that included recruitment bias, non‐response, ascertainment of illicit drug use, ascertainment of health‐care utilization, adequacy of follow‐up (for cohort studies), selection of comparison groups (for relative measures) and adjustment (for relative measures). Full details are given in Supporting information.

### Screening and data extraction

Two authors (D.L. and J.F.) independently screened titles and abstracts using Rayyan 20. There was agreement of 94% (Cohen's kappa 0.58) and conflicts were resolved through discussion. We accessed full texts, and one author (D.L., J.F. or E.K.) used a piloted data extraction tool to record details including the study design, year, location of the study, recruitment setting (drug treatment services, community or health care), participant demographics, predominant drugs used and denominator and numerator for primary outcomes. Where relative frequencies (such as rate ratios) were reported, we also recorded the ratio and details of the comparison group. Where predictors of health‐care use and cause‐specific health‐care use were reported, we marked the study for narrative synthesis. A second author checked that all data was accurate. Queries that could not be resolved were referred to K.I.M. for a final decision.

### Analysis

In a narrative review, we described: (i) the range of values of the primary outcomes; (ii) predictors of health‐care utilization; and (iii) causes of health‐care utilization by disease.

In quantitative analysis, we displayed frequency rates of ED and in‐patient utilization using forest plots. To provide informal comparisons with the general population, we used published frequencies of health‐care utilization in the United States, Canada, Australia and the United Kingdom 21, 22, 23 for the general population group with the most similar age and sex profile as the study population. Details of the comparison group used for each study are given in the archived data set.

We conducted a random‐effects meta‐analysis to report the average frequency of health‐care utilization across study populations, limited to results from high‐income countries, and excluded studies of subgroups likely to have unusual health‐care utilization (such as people living with HIV and prisoners). We anticipated that the strongest determinants of heterogeneity would be the predominant drug and the country where the study was conducted, and therefore stratified results by these variables. As an exploratory analysis of further sources of heterogeneity (not pre‐specified), we included each of the following variables in the meta‐analysis equation as a moderator 24: recruitment setting (health care, drug treatment services, community or prison), country, study design, study era (1990–99, 2000–09, 2010–18), risk‐of‐bias score (low or high), age (average age under or over 30 years) and sex (greater or less than 60% male), using a threshold of *P* < 0.05 to identify significant moderators.

All analysis was conducted using R version 3.5.1.

## Results

### Search results

**Figure 1:**
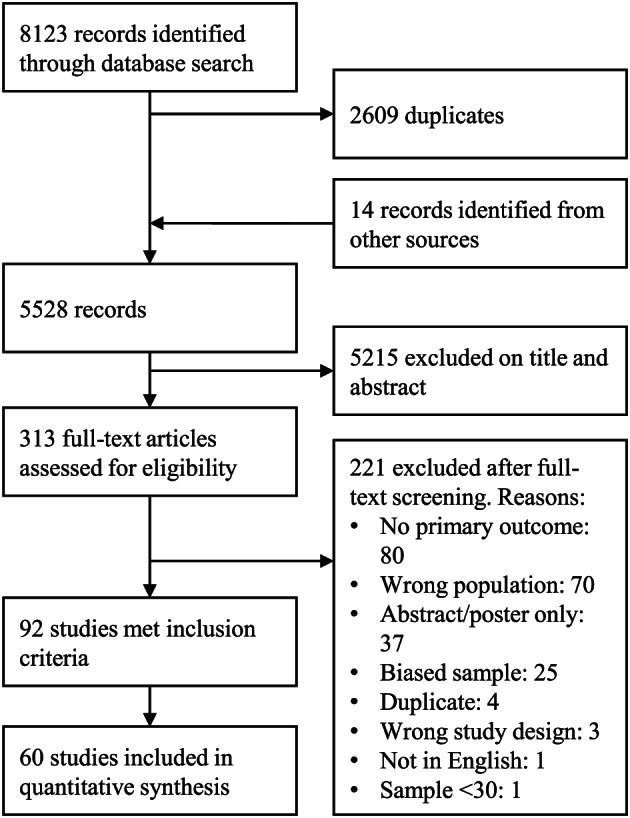
Flow‐chart of included studies

Our search identified 5528 studies after de‐duplication, 313 of which were selected for full‐text review, and 92 were included. Figure 1 shows a flow‐chart of studies. Some studies included groups from distinct regions or with distinct drug use patterns, while others duplicated samples from other studies, and we identified 98 unique populations with 204 relevant data points. The full data set is available in Supporting information.

### Description of study populations

Of the 98 study populations, 53 were in the United States; 16 in Australia; 13 in Canada; three in Ireland; two each in Taiwan, Italy, New Zealand, United Kingdom, Vietnam; and one each in Denmark, Finland and Norway.

**Figure 2:**
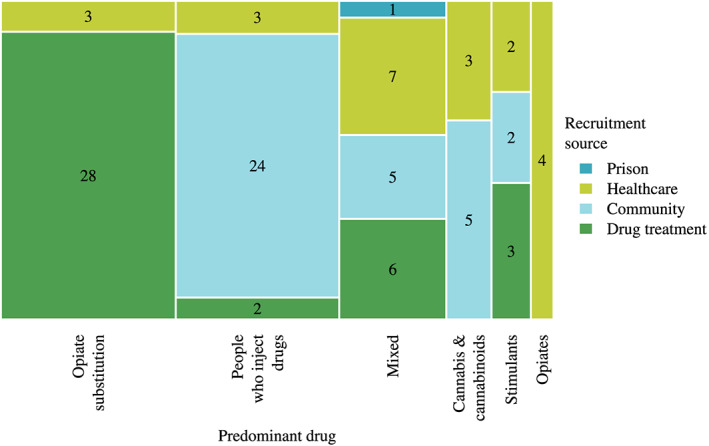
Unique study populations by predominant drug and recruitment source [Colour figure can be viewed at wileyonlinelibrary.com]

Although the search strategy included people using any illicit drugs, studies focused on people who used illicit drugs associated with dependence. The largest group was people using opiate substitution (31 populations), mainly recruited from drug treatment services. The next largest comprised people who inject drugs (29 populations), mainly recruited from community settings. Eight studies focused on cannabis users, seven focused on stimulant users (where injecting was not specified) and five focused on opiate users (where injecting was not specified). Figure 2 shows the number of study populations by predominant drug used and recruitment setting. No studies recruited participants who predominantly used MDMA/ecstasy, powder cocaine, novel psychoactive substances or hallucinogens such as lysergic acid diethylamide (LSD) and psilocybin.

A mean of 68% [standard deviation (SD) = 12%] of participants were male and the mean of average ages (reported in some studies as means and in others as medians) was 36.7 (SD = 6.0).

### Study quality

Fifty‐eight of 204 data points had high risk of bias. The main risk was lack of information on non‐response. The overall risk of bias was not associated with frequency of health‐care utilization in meta‐analysis (see below). Table 1 summarizes results from the quality assessment.

**Table 1:** Results of quality assessment.

	Data points	High risk	Proportion high risk
Recruitment bias	204	28	14%
Non‐response	204	121	59%
Ascertainment of illicit drug use	204	43	21%
Ascertainment of health‐care utilization	204	44	22%
Adequacy of follow‐up	82	21	26%
Selection of comparison group	47	4	9%
Adjustment for confounders	47	4	9%
Global assessment	204	58	28%

### Narrative review

#### Range of values

Frequencies of all outcomes were high and heterogeneous. ED utilization ranged from 19 25 to 1061 26 per 100 person‐years. The proportion of participants visiting ED in the past 12 months ranged from 10% 27 to 72% 28. Studies including relative measures showed frequency of ED utilization of three to 10 times that of comparison groups not using illicit drugs 29, 30, 31, 32. Exceptions were a study in rural Taiwan, showing that people who inject heroin had a similar rate of ED presentation as the general population 33, and a study of older people who use cannabis in the United States showing similar odds of ED presentation as those who do not use cannabis 34.

The rate of in‐patient episodes ranged from 8 33 to 852 29 per 100 person‐years. The proportion of participants who were hospitalized during the past 12 months ranged from 8% 35 to 41% 36. Studies including relative measures showed frequency of hospital admission two to eight times that of comparison groups not using illicit drugs 29, 30, 31, 37, 38, 39, 40. Again, studies of people who inject drugs in rural Taiwan and older people who use cannabis in the United States were exceptions, showing similar frequencies of hospital admission to the general population 33, 41.

There were fewer studies of primary care utilization. Ten studies reported rates, ranging from 231 42 to 2087 37 episodes per 100 person‐years. The proportion of participants visiting primary care in the past 12 months ranged from 38% 43 to 90% 44. Three studies found a higher frequency than the general population: a study of insurance data in Canada found people with diagnoses of ‘substance abuse’ had 4.2 times more primary care visits than those without this diagnosis 37; a study of patients at a specialist primary care clinic in Ireland that found that those with methadone prescriptions had 4.2 times the odds of a primary care consultation during 6 months, excluding visits for drug‐related problems 45; and a study of people in drug treatment in Australia that found those primarily in treatment for opioids had a median of 12 primary care visits in the past year, compared to seven for those in treatment for alcohol 44. Other studies found a low absolute frequency of presentation without providing formal comparisons with the general population. For example, only 58% of people who inject drugs in Baltimore saw a primary care doctor over 3 years 46; 53% of people who use methamphetamine in Australia saw a primary care doctor over 12 months 47; and 32% of people who inject drugs in Montreal saw a primary care provider over 6 months, which was informally compared to 90% in the general population 48.

Studies investigating the frequency of health‐care utilization in more than one setting showed that primary care episodes are more frequent than ED or in‐patient episodes 49, 50, 51, 52, 53.

#### Predictors of health‐care utilization

ED presentation was consistently associated with regular or recent injecting 54, 55, 56, 57, sex work, 54, 58 diagnosed hepatitis C 40, diagnosed HIV 31, 36, 56, 59, 60, female sex 36, 49, 61, 62, 63, 64, homelessness or unstable housing 26, 55, 56, 61, 65, crack cocaine or stimulant use 56, 61, 62, alcohol use 63, 66, 67, polydrug use 47, 68 and mental health problems 36, 37, 63.

Hospital admission was associated with similar factors: regular or recent injecting 55, 56, 57, 69, 70, diagnosed hepatitis C 71, 72, diagnosed HIV 35, 56, 69, 70, 73, low CD4 count among HIV‐positive participants 74, female sex 38, 39, 49, 69, 70, 72, 74, homelessness or unstable housing 55, 69, alcohol use 72, polydrug use 47 and mental health problems 31, 37.

One study (the Melbourne Injecting Drug User Cohort Study) reported similar associations with primary care utilization: regular injecting, homelessness, cocaine injection and unstable income 48, 75.

Opiate substitution treatment was consistently associated with a lower frequency of ED presentation and hospital admission 27, 36, 42, 53, 57, 71, 73, 76, 77, 78, 79, 80, 81 than comparison groups of untreated opiate users. Among substitution patients, consistent medication was associated with a lower rate of ED utilization 77, 78, 82. Some studies looked at different types of treatment. For example, one study found that take‐home methadone was associated with a lower risk of hospital admission 83. No studies looked at the effect of treatment for dependence on drugs other than opiates.

Some studies reported non‐significant associations with these factors, but none found effects in the opposite direction.

Although some studies show that mental or physical morbidity predicts health‐care utilization, no studies attempted to show whether increased frequency of health‐care utilization among people who use illicit drugs was explained by morbidity or other indicators of need for services.

#### Causes of health‐care utilization

**Figure 3:**
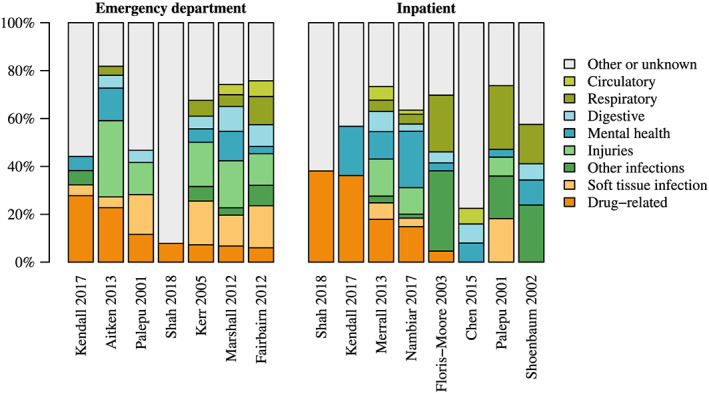
Main reason for health‐care utilization [Colour figure can be viewed at wileyonlinelibrary.com]

Studies with cause‐specific data showed that a minority of ED and in‐patient episodes relate to the direct effects of illicit drugs, such as withdrawal, overdose and intoxication (Fig. 3). Infections and particularly skin and soft‐tissue infections were common causes of ED and in‐patient episodes in study populations in Canada 26, 31, 54, 56, 59, 69, Norway 42 and Taiwan 33. All infections, and particularly pneumonias, were important causes of health‐care utilization in HIV‐positive opiate users 70, 74. Infections were less important causes of health‐care utilization in Australia 84, 85. Traumas, injuries and mental health problems were important causes of ED utilization and hospital admission in all countries 33, 54, 56, 72, 84, 85.

### Quantitative analysis

We conducted a meta‐analysis of health‐care utilization rates (25 studies reporting ED episodes and 25 reporting hospital admission) and 12‐month cumulative incidence (11 studies reporting ED episodes and 11 reporting hospital admission). Twelve months was the most common period examined in the literature. While we collected data from studies of other periods, we did not analyse these data because the periods varied too widely. We were unable to determine the consistency of the definition of primary care visits among studies and therefore did not attempt quantitative analysis. We restricted the analysis to populations who primarily use heroin, crack cocaine or methamphetamine or have a diagnosis of ‘substance abuse disorder’ or drug dependence, as there were few studies of people who use cannabis or have other patterns of use.

**Figure 4:**
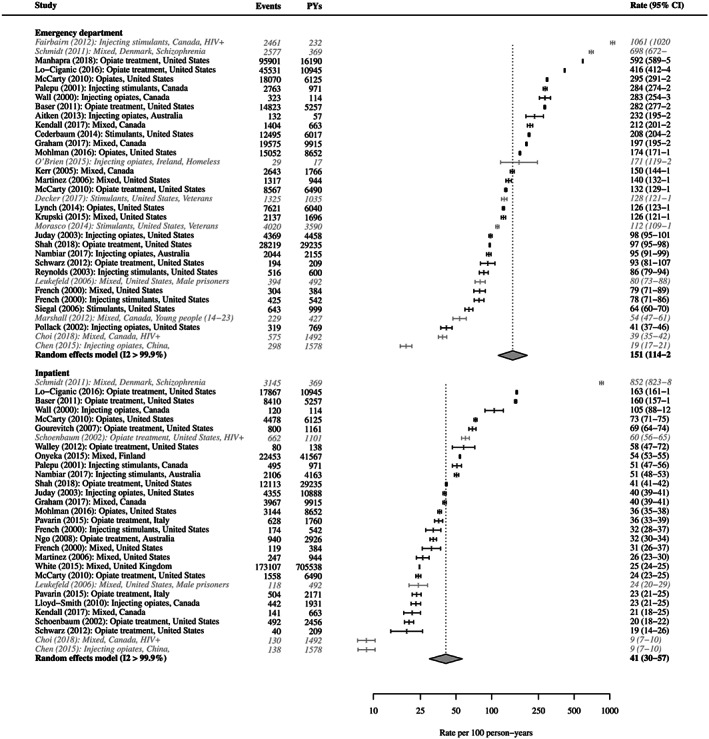
Forest plot of rates of health‐care utilization. Studies in grey and italics are not included in the pooled estimate

**Figure 5:**
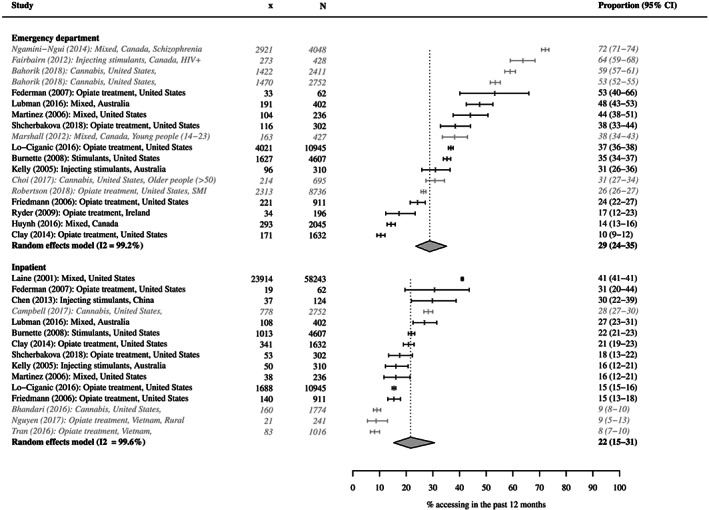
Forest plot of 12‐month cumulative incidence of health‐care utilization. Studies in grey and italics are not included in the pooled estimate

ED frequencies are shown in Figs 4 and 5. An average of 29% [95% confidence interval (CI) = 24–35%] of participants visited ED over a 12‐month period. The pooled rate was 151 visits per 100 person‐years (95% CI = 114–201). There was high heterogeneity, with *I* ^2^ approaching 100% for both analyses. Thirty‐two study populations were matched with published rates for groups of a similar age and sex in the general population. ED presentation ranged from 0.9 to 24.7 times the general population (mean 4.8). Stratified meta‐analysis by predominant drug and country did not show significant differences to the overall pooled estimate (see Supporting information), and the exploratory meta‐regression found no significant moderators.

Hospital admission rates and cumulative incidences are shown in Figs 4 and 5. An average of 22% (95% CI = 15–31%) of participants were hospitalized over a 12‐month period. The pooled rate was 41 episodes per 100 person‐years (95% CI = 30–57). There was high heterogeneity, with *I* ^2^ approaching 100% for both analyses. Twenty‐seven study populations were matched with published rates for comparable groups in the general population. Hospital admission rates ranged from 1.9 to 35.5 times the general population (mean 7.1). As with the ED results, stratified meta‐analysis by predominant drug and country did not show significant differences to the overall pooled estimate, and the exploratory meta‐regression found no significant moderators.

## Discussion

To our knowledge, this is the first systematic review of health‐care utilization in people who use illicit drugs. The majority of available evidence relates to people who use heroin, methamphetamine and crack cocaine or have a diagnosis of drug dependence. The results show high but widely varying frequencies of ED presentation and hospital admission in this group.

The pooled frequencies of ED and hospital admissions are substantially higher than the general population. In part, this reflects morbidity and greater need for treatment. However, higher utilization does not necessarily represent good health‐care access. A systematic review in 2009 86 identified 10 studies showing that people with substance use disorders are less likely to receive definitive treatment for specific conditions, despite higher all‐cause attendance. For example, a study of veterans with diagnoses of diabetes in the United States found that participants with comorbid substance use were less likely to receive foot or retina examinations 87. Our finding of high utilization of acute services may not represent good access, but a pattern where primary and preventative health care is poor and unplanned health care is common.

The results contrast with studies of health care among people who use alcohol, which find that drinkers (including heavy drinkers) have lower rates of health‐care utilization than abstainers 88. This is likely to be explained by abstention among people who are unwell, rather than a protective effect of alcohol. In contrast, this review found that people who use illicit drugs present to health services much more frequently than the general population. This may be because studies of people who use illicit drugs tend to focus on people who are dependent on or use drugs associated with health harms, while studies of alcohol may include more moderate drinkers.

Predictors of health‐care utilization were consistent across studies, including unstable housing, drug injection and mental health problems. These factors reflect previously identified risk factors for poor health in people who use drugs 89, and are likely to be associated with greater need for health care.

Effectively, all the variation across studies was due to differences between populations rather than within‐study error. Despite consistent predictors of health‐care utilization within studies, we were not able to explain the variation between studies by the predominant drugs used by study participants, the country of the study or any other study‐level variables that we collected. Results varied widely even within countries and populations with apparently similar drug use. For example, in the United States, the rate of hospital admission of people in opiate substitution therapy ranged from 51 to 592 per 100 person‐years 53, 76, 77, 78, 90, 91, 92. Other research has conceptualized access to health services as a product of individual factors, social contexts and health‐care systems 93, 94. The extent of the heterogeneity in our results is unlikely to be fully explained by individual‐level factors that we did not capture. This suggests that social and health‐care contexts can substantially affect health‐care utilization. The heterogeneity also highlights the difficulty of generalizing results from single studies of health‐care utilization.

The review identified three main gaps in the evidence. First, 84% of study populations were from the United States, Canada or Australia. We did not identify any studies from low‐income countries. Secondly, there were few studies with primary care data, even though existing studies suggest people who use illicit drugs visit primary care more often than acute health‐care settings 49, 50, 51, 52, 53, contrary to the stereotype of reliance on ED. Thirdly, almost all studies were of people who use heroin, crack cocaine or methamphetamine or have a diagnosis of drug dependence. There were only eight studies of people who use cannabis and none of people using MDMA/ecstasy, powder cocaine, hallucinogens, novel psychoactive substances or other drugs.

The results highlight the need for interventions that improve general health outcomes among people who use drugs. Despite a body of research into the effectiveness of opiate substitutes to reduce use of street heroin 95, community‐distributed naloxone to prevent overdose deaths 96, strategies to reduce transmission of hepatitis C and improve access to hepatitis C treatment 97 and some strategies to improve treatment of soft‐tissue infections among people who inject drugs 98, there is limited research into interventions that can improve treatment of health problems that are not specifically associated with drug use. Some studies have shown that Housing First can reduce all‐cause ED utilization, although study outcomes tend to focus on substance use rather than broader health 99. Case management (where a single case manager is assigned to each patient) can improve drug treatment outcomes but, again, evidence of the effect on broader health outcomes is limited 100.

### Limitations of the evidence

Most studies in the past have described patients in health‐care services to show the proportion that use drugs, rather than using population‐based approaches. This has led in particular to a focus on ED and frequent healthcare users. To broaden this focus, we synthesized observational studies that often report health‐care utilization as a secondary outcome. The strength of this approach is that it has shown the wide variation in utilization of acute hospital services, and in some settings primary care may be attended more frequently. The limitation is that many studies provide limited insight into predictors and patterns of utilization.

Half the studies in the review (43 of 92) rely on linked electronic health‐care records, which may have inaccuracies in diagnostic coding. For example, there is evidence that drug‐related events such as overdoses are under‐recorded in ED data and may be given other diagnostic codes 101, 102. This could contribute to the small proportion of health‐care episodes that are ‘drug‐related’ in our results. In addition, few studies include data from the recent period when synthetic opioids such as fentanyl became more common in North American illicit drug markets. Opioid‐related overdoses in the United States have increased during this period 103, and the proportion of health‐care episodes that are drug‐related may have increased.

The quality assessment identified non‐response as the most common problem. This usually resulted from recruitment relying on volunteers or convenience samples rather than a systematic or random approach. These methods are often necessary, as it can be difficult to construct sample frames of people who use drugs. Difficulties in constructing sample frames may also account for the relative lack of studies of people using some illicit drugs, such as powder cocaine, although this may also be due to less severe health outcomes in these groups.

None of the studies included in this review looked at whether higher morbidity explained higher rates of health‐care use, so we were not able to discuss the appropriateness of health service use.

### Limitations of the review and meta‐analysis

First, we only included English‐language studies, which may partially explain the large proportion of studies from English‐speaking countries—although the English‐language restriction only Excluded 179 of 5528 search results. Secondly, given the heterogeneity of results, meta‐analysis is only intended to provide an average across studies, rather than a meaningful estimate of health‐care utilization for any specific population. Thirdly, we defined health‐care utilization with simple rates or proportions. While this enabled us to perform a traditional systematic review, it meant that the results provide limited insight into the appropriateness or equity of the high rates of health‐care utilization that we observed. Finally, our review focused on three mainstream health‐care settings (primary care, ED and in‐patient hospital care), and did not consider other potential sources of health care such as community drug treatment services, which sometimes provide a wider set of interventions. Future research should consider the full range of health‐care provision for people who use drugs, including opportunities for integration between drug treatment and mainstream health services.

## Conclusion

People who use illicit drugs present to acute health services several times more often than comparison groups throughout primary care, ED and in‐patient settings, reflecting high morbidity. Utilization rates are highest in those who inject drugs, homeless people and those with mental health problems. Research is needed into the quality of health care for people who use illicit drugs, provision of health care in non‐acute settings and the development of health services that are considered safe and acceptable to this group.

### Declaration of interests

T.M. has been a member of the organizing committee for, and chaired, conferences supported by unrestricted educational grants from Reckitt Benckiser, Lundbeck, Martindale Pharma and Britannia Pharmaceuticals Ltd (unpaid). He collaborates (unpaid) with and has received research funding from the substance misuse treatment provider Change Grow Live. He has received speaker honoraria from a commercial health‐care consultancy firm (Applied Strategic). D.C. reports personal fees from Mallinckrodt Pharmaceuticals and personal fees from Nektar Therapeutics, outside the submitted work.

## Supporting information

Data S1. Supporting information.
