# Supplementary material for: Frequency of health‐care utilization by adults who use illicit drugs: a systematic review and meta‐analysis
Source: Addiction. 2020 Feb 10;115(6):1011–23. doi: 10.1111/add.14892 (PMC7210080; doi:10.1111/add.14892)
Supplement: Supplementary file 1 — Data S1. Supporting information. [file ADD-115-1011-s001.pdf]

Supplementary Information

1. Search terms
2. Modified Newcastle-Ottowa Scale
3. Full list of included studies
4. Stratified forest plots
5. PRISMA checklist

1 Search terms

Medline

1 addict*.mp
2 (chemical adj2 dependenc*).mp
3 (substance adj2 misuse*).mp
4 (substance adj2 abus*).mp
5 substance use.mp
6 drug adj1 user*.mp
7 (drug adj2 abus*).mp
8 (drug adj2 dependen*).mp
9 (inject* adj2 drug*).mp
10 heroin.mp
11 opiate*.mp
12 cocaine.mp
13 crack.mp
14 amphetamine*.mp
15 methamphetamine.mp
16 benzodiazepine.mp
17 mdma.mp
18 ecstasy.mp
19 cannabis.mp
20 Substance-Related Disorders/
21 Amphetamine-Related Disorders/
22 Cocaine-Related Disorders/
23 Heroin Dependence/
24 Substance Abuse, Intravenous/
25 Cannabis/
26 Marijuana abuse/
27 Heroin/
28 Crack Cocaine/
29 Cocaine/
30 Methamphetamine/
31 Amphetamine/
32 Benzodiazepines/
33 or/1-32
34 healthcare use.mp
35 healthcare usage.mp
36 care use.mp
37 care usage.mp
38 service use.mp
39 service usage.mp
40 (hospital* adj3 rate*).mp
41 (hospital* adj3 incidence).mp
42 (hospital* adj3 prevalence).mp
43 (”use of” adj2 primary).mp
44 (”use of” adj2 secondary).mp
45 (”use of” adj2 emergency).mp
46 (”use of” adj2 service*).mp
47 (”use of” adj2 healthcare).mp
48 (”use of” adj2 care).mp
49 (utili* adj2 primary).mp

50 (utili* adj2 secondary).mp
51 (utili* adj2 emergency).mp
52 (utili* adj2 service*).mp
53 (utili* adj2 healthcare).mp
54 (utili* adj2 care).mp
55 (visits adj2 primary).mp
56 (visits adj2 secondary).mp
57 (visits adj2 emergency).mp
58 (visits adj2 service*).mp
59 (visits adj2 healthcare).mp
60 (visits adj2 care).mp
61 Health Resources/
62 Health Expenditures/
63 Primary Health Care/
64 Secondary Care/
65 or/34-64
66 Epidemiologic studies/
67 exp cohort studies/
68 (cohort adj1 stud*).tw.
69 (cohort adj1 analy*).tw.
70 (”follow up” adj1 stud*).tw
71 (observational adj1 stud*).tw.
72 Longitudinal.tw
73 Retrospective.tw.
74 cross-sectional.tw.
75 Cross-sectional studies/
76 Surveys and Questionnaires/
77 linkage.tw
78 survey.tw
79 or/66-78
80 33 and 65
81 79 and 80
82 limit 81 to (english language and yr=”2000 -Current”)

Embase

1 addict*.mp
2 (chemical adj2 dependenc*).mp
3 (substance adj2 misuse*).mp
4 (substance adj2 abus*).mp
5 substance use.mp
6 drug adj1 user*.mp
7 (drug adj2 abus*).mp
8 (drug adj2 dependen*).mp
9 (inject* adj2 drug*).mp
10 heroin.mp
11 opiate*.mp
12 cocaine.mp
13 crack.mp
14 amphetamine*.mp
15 methamphetamine.mp
16 benzodiazepine.mp

17 mdma.mp
18 ecstasy.mp
19 cannabis.mp
20 addiction/
21 drug dependence/
22 amphetamine dependence/
23 cannabis addiction/
24 opiate addiction/
25 cocaine dependence/
26 methamphetamine dependence/
27 heroin dependence/
28 drug misuse/
29 heroin/
30 cannabis/
31 cocaine/
32 amphetamine/
33 methamphetamine/
34 or/1-33
35 healthcare use.mp
36 healthcare usage.mp
37 care use.mp
38 care usage.mp
39 service use.mp
40 service usage.mp
41 (hospital* adj3 rate*).mp
42 (hospital* adj3 incidence).mp
43 (hospital* adj3 prevalence).mp
44 (”use of” adj2 primary).mp
45 (”use of” adj2 secondary).mp
46 (”use of” adj2 emergency).mp
47 (”use of” adj2 service*).mp
48 (”use of” adj2 healthcare).mp
49 (”use of” adj2 care).mp
50 (utili* adj2 primary).mp
51 (utili* adj2 secondary).mp
52 (utili* adj2 emergency).mp
53 (utili* adj2 service*).mp
54 (utili* adj2 healthcare).mp
55 (utili* adj2 care).mp
56 (visits adj2 primary).mp
57 (visits adj2 secondary).mp
58 (visits adj2 emergency).mp
59 (visits adj2 service*).mp
60 (visits adj2 healthcare).mp
61 (visits adj2 care).mp
62 hospital utilization/
63 health care utilization/
64 or/35-63
65 Longitudinal study/
66 Retrospective study/
67 Randomized controlled trials/
68 66 not 67
69 Cohort analysis/
70 (cohort adj1 stud*).mp

71 (”follow up” adj1 stud*).tw
72 (observational adj1 stud*).tw.
73 (epidemiologic* adj1 stud*).tw.
74 Longitudinal.tw
75 Retrospective.tw.
76 cross-sectional.tw.
77 linkage.tw
78 survey.tw
79 or/65-78
80 34 and 64
81 79 and 80
82 limit 81 to (english language and yr=”2000 -Current”)

PsychINFO

1 addict*.mp
2 (chemical adj2 dependenc*).mp
3 (substance adj2 misuse*).mp
4 (substance adj2 abus*).mp
5 substance use.mp
6 drug adj1 user*.mp
7 (drug adj2 abus*).mp
8 (drug adj2 dependen*).mp
9 (inject* adj2 drug*).mp
10 heroin.mp
11 opiate*.mp
12 cocaine.mp
13 crack.mp
14 amphetamine*.mp
15 methamphetamine.mp
16 benzodiazepine.mp
17 mdma.mp
18 ecstasy.mp
19 cannabis.mp
20 Addiction/
21 Drug Abuse/
22 Drug Dependency/
23 Drug Addiction/
24 Substance Use Disorder/
25 Heroin Addiction/
26 Heroin/
27 Opiates/
28 Cocaine/
29 Crack Cocaine/
30 Amphetamine/
31 Methamphetamine/
32 Methylenedioxymethamphetamine/
33 Cannabis/
34 or/1-33
35 healthcare use.mp
36 healthcare usage.mp
37 care use.mp

38 care usage.mp
39 service use.mp
40 service usage.mp
41 (hospital* adj3 rate*).mp
42 (hospital* adj3 incidence).mp
43 (hospital* adj3 prevalence).mp
44 (”use of” adj2 primary).mp
45 (”use of” adj2 secondary).mp
46 (”use of” adj2 emergency).mp
47 (”use of” adj2 service*).mp
48 (”use of” adj2 healthcare).mp
49 (”use of” adj2 care).mp
50 (utili* adj2 primary).mp
51 (utili* adj2 secondary).mp
52 (utili* adj2 emergency).mp
53 (utili* adj2 service*).mp
54 (utili* adj2 healthcare).mp
55 (utili* adj2 care).mp
56 (visits adj2 primary).mp
57 (visits adj2 secondary).mp
58 (visits adj2 emergency).mp
59 (visits adj2 service*).mp
60 (visits adj2 healthcare).mp
61 (visits adj2 care).mp
62 Primary Health Care
63 Health Care utilization
64 or/35-63
65 Longitudinal Studies/
66 Followup Studies/
67 Retrospective Studies/
68 Cohort analysis/
69 (cohort adj1 stud*).tw.
70 (”follow up” adj1 stud*).tw.
71 (observational adj1 stud*).tw.
72 (epidemiologic* adj1 stud*).tw.
73 Longitudinal.tw.
74 Retrospective.tw.
75 Cross sectional.tw.
76 cross-sectional.tw.
77 linkage.tw.
78 survey.tw.
79 or/65-78
80 34 and 64
81 79 and 80
82 limit 80 to (english language and yr=”2000 -Current”)

2 Modified Newcastle-Ottowa Scale

Representativeness of people who use illicit drugs

Good representativeness of target population (e.g. random sampling, complete sample)	1
Selection process does not ensure representativeness (e.g. snowball sampling, systematic sample), but is clearly described and is unlikely to select low or high healthcare users	1
Selection process likely to select low or high healthcare users	0
No description of the derivation of sample or unclear	0

Non-response

Comparability between respondents and non-respondents was established and/or response rate >=70%	1
The comparability between respondents and non-respondents was unsatisfactory and response rate <70% (or not specified)	0
No statement or unclear	0

Ascertainment of illicit drug use

From structured interview or medical records: clear description of drugs used	1
From structured interview or medical records: partial description of drugs used	1
Not directly ascertained (e.g. relying drug-related sampling locations)	0
No statement or unclear	0

Ascertainment of healthcare use

Record linkage	1
Self report: questions described and consistently applied	1
Self report: questions not described or not consistently applied	0
No statement or unclear	0

Adequacy of follow-up (cohorts only)

Complete follow up - all subjects accounted for	1
Subjects lost to follow up unlikely to introduce bias - small number lost (>=80% follow up) or description provided of those lost showing they are similar	1
Follow up rate <80% and no description of those lost (or description shows they are substantially different)	0
No statement or unclear	0

Selection of the comparison group, for studies with comparative measures (e.g. rate ratios, prevalence ratios)

Drawn from the same population that people who use drugs are drawn from	1
Drawn from a different source	0
No statement or unclear	0

Comparability of groups on the basis of design or analysis, for studies with comparative measures (e.g. rate ratios, prevalence ratios)

Study controls for a number of confounders, including the most important (likely to be age)	1
Study controls for the most important confounder only	1
Study controls for confounders, but not the most important one	0
No control for differences between groups	0

Determining overall risk of bias

Each study has a certain number of points ’available’, depending on the study design and if comparative measures are used. Points are totalled and then summarised as low or high risk using the table below.

Maximum points available	High risk band
4	0-2
5	0-3
6	0-3
7	0-4

3 Full list of included studies

First author	Title	Year	Journal	Volume	Issue
French	Chronic illicit drug use, health services utilization and the cost of medical care	2000	Soc Sci Med	50	0
Wall	Social costs of untreated opioid dependence	2000	Journal of urban health	77	4
Knowlton	Access to medical care and service utilization among injection drug users with HIV/AIDS	2001	Drug Alcohol Depend	64	0
Laine	Regular outpatient medical and drug abuse care and subsequent hospitalization of persons who use illicit drugs	2001	Journal of the American Medical Association	285	18
Palepu	Hospital utilization and costs in a cohort of injection drug users.	2001	CMAJ	165	4
Pollack	The impact of needle exchange-based health services on emergency department use	2002	Journal of General Internal Medicine	17	5
Riley	Health services utilization by injection drug users participating in a needle exchange program	2002	Am J Drug Alco- hol Abuse	28	3
Schoenbaum	Predictors of hospitalization for HIV-positive women and men drug users, 1996-2000.	2002	Public health reports	117	NA
Darke	Health service utilization and benzodiazepine use among heroin users: Findings from the Australian Treatment Outcome Study (ATOS)	2003	Addiction	98	8
Floris-Moore	Gender and hospitalization patterns among HIV-infected drug users before and after the availability of highly active antiretroviral therapy.	2003	Journal of acquired immune deficiency syndromes	34	3
Juday	The role of Medicaid HMO enrollment in the longitudinal utilization of medical care services in a cohort of injecting drug users in Baltimore, Maryland.	2003	Substance Abuse	24	1
Reynolds	Use of emergency room services by out-of-treatment drug users in Long Beach, California	2003	Journal of Addictive Diseases	22	2
Robles	Determinants of health care use among Puerto Rican drug users in Puerto Rico and New York City.	2003	Clinical infectious diseases	37	12
Stein	Injection frequency mediates health service use among persons with a history of drug injection	2003	Drug and Alcohol Dependence	70	2
Turner	Effects of long-term, medically supervised, drug-free treatment and methadone maintenance treatment on drug users emergency department use and hospitalization	2003	Clinical Infectious Diseases	37	S5
Wang	Mortality in HIV-seropositive versus -seronegative persons in the era of highly active antiretroviral therapy: implications for when to initiate therapy.	2004	The Journal of infectious diseases	190	6
Kelly	Health service utilisation among regular methamphetamine users	2005	NDARC Technical Report	NA	0
Kerr	High rates of primary care and emergency department use among injection drug users in Vancouver	2005	Journal of Public Health	27	1

First author	Title	Year	Journal	Volume	Issue
Lundgren	Factors associated with emergency room use among injection drug users of African-American, Hispanic and White-European background	2005	Am J Addict	14	3
Mills	Post-traumatic stress disorder among people with heroin dependence in the Australian treatment outcome study (ATOS): prevalence and correlates.	2005	Drug and Alcohol Dependence	77	3
Friedmann	Do Mechanisms that Link Addiction Treatment Patients to Primary Care Influence Subsequent Utilization of Emergency and Hospital Care?	2006	Medical care	44	1
Leukefeld	A prospective examination of high-cost health services utilization among drug using prisoners reentering the community.	2006	The journal of behavioral health services & research	33	1
Martinez	Impact of Permanent Supportive Housing on the Use of Acute Care Health Services by Homeless Adults.	2006	Psychiatric Services	57	7
Siegal	Emergency department utilization by crack-cocaine smokers in dayton, ohio	2006	Am J Drug Alcohol Abuse	32	1
Darke	Changes in the use of medical services and prescription drugs amongst heroin users over two years	2007	Drug Alcohol Rev	26	0
Federman	Primary care affiliations of adults in a methadone program with onsite care	2007	Journal of Addictive Diseases	26	1
Gourevitch	On-site medical care in methadone maintenance: associations with health care use and expenditures.	2007	Journal of substance abuse treatment	32	2
Baum	Quality of life, symptomatology and healthcare utilization in HIV/HCV co-infected drug users in Miami.	2008	Journal of addictive diseases	27	2
Burnette	Prevalence and health correlates of prostitution among patients entering treatment for substance use disorders.	2008	Archives of General Psychiatry	65	3
Ngo	Comparing drug-related hospital morbidity following heroin dependence treatment with methadone maintenance or naltrexone implantation.	2008	Archives of General Psychiatry	65	4
Skeie	Somatic health among heroin addicts before and during opioid maintenance treatment: A retrospective cohort study	2008	BMC Public Health	8	NA
Benjamin-Johnson	Access to medical care, use of preventive services, and chronic conditions among adults in substance abuse treatment	2009	Psychiatric Services	60	12
Cullen	Chronic illness and multimorbidity among problem drug users: A comparative cross sectional pilot study in primary care	2009	BMC family practice	10	NA
Ryder	Prevalence of problem alcohol use among patients attending primary care for methadone treatment	2009	BMC family practice	10	NA
Hartzler	Dissolution of a harm reduction track for opiate agonist treatment: Longitudinal impact on treatment retention, substance use and service utilization.	2010	International Journal of Drug Policy	21	1

First author	Title	Year	Journal	Volume	Issue
Lloyd-Smith	Determinants of hospitalization for a cutaneous injection-related infection among injection drug users: a cohort study	2010	BMC public health	10	NA
McCarty	Methadone maintenance and the cost and utilization of health care among individid- uals dependent on opioids in a commercial health plan	2010	Drug and Alcohol Dependence	0	0
Robbins	Health and oral health care needs and health care-seeking behavior among homeless injection drug users in San Francisco.	2010	Journal of urban health	87	6
Baser	Cost and utilization outcomes of opioid-dependence treatments	2011	The American journal of managed care	17	NA
Schmidt	The impact of substance use disorders on the course of schizophrenia-A 15-year follow-up study: Dual diagnosis over 15 years.	2011	Schizophrenia Research	130	1
Fairbairn	Emergency department utilization among a cohort of HIV-positive injecting drug users in a Canadian setting	2012	The Journal of emergency medicine	43	2
Marshall	Frequent methamphetamine injection predicts emergency department utilization among street-involved youth.	2012	Public Health	126	1
Schwarz	Retention on buprenorphine treatment reduces emergency department utilization, but not hospitalization, among treatment-seeking patients with opioid dependence.	2012	Journal of substance abuse treatment	43	4
Walley	Methadone dose, take home status, and hospital admission among methadone maintenance patients.	2012	Journal of Addiction Medicine	6	3
Aitken	A cross-sectional study of emergency department visits by people who inject drugs	2013	Emergency medicine journal	30	5
Chen	Health care service utilization and associated factors among heroin users in Northern Taiwan.	2013	Addictive Behaviors	38	11
Dietze	The relationship between alcohol use and injecting drug use: Impacts on health, crime and wellbeing	2013	Drug and Alcohol Dependence	128	1
Horyniak	Establishing the Melbourne Injecting Drug User Cohort Study (MIX): Rationale, methods, and baseline and twelve-month follow-up results.	2013	Harm Reduction Journal	10	1
Mark	Psychiatric and medical comorbidities, associated pain, and health care utilization of patients prescribed buprenorphine.	2013	Journal of substance abuse treatment	44	5
Merrall	A record linkage study of hospital episodes for drug treatment clients in Scotland, 1996-2006.	2013	Addiction Research & Theory	21	1
Cederbaum	Utilization of emergency and hospital services among individuals in substance abuse treatment.	2014	Substance Abuse Treatment, Prevention, and Policy	9	NA
Clay	Persistence and healthcare utilization associated with the use of buprenorphine/naloxone film and tablet formulation therapy in adults with opioid dependence	2014	Journal of Medical Economics	17	9

First author	Title	Year	Journal	Volume	Issue
Fuster	No detectable association between frequency of marijuana use and health or healthcare utilization among primary care patients who screen positive for drug use.	2014	Journal of General Internal Medicine	29	1
Lynch	Costs of care for persons with opioid dependence in commercial integrated health systems.	2014	Addiction science & clinical practice	9	NA
Morasco	Comparison of health service use among veterans with methamphetamine versus alcohol use disorders.	2014	Journal of addiction medicine	8	1
Nambiar	A cross-sectional study describing factors associated with utilisation of GP services by a cohort of people who inject drugs	2014	BMC health services research	14	NA
Ngamini-Ngui	High users of emergency departments in quebec among patients with both schizophrenia and a substance use disorder	2014	Psychiatric Services	65	11
Artenie	Visits to primary care physicians among persons who inject drugs at high risk of hepatitis C virus infection: Room for improvement	2015	Journal of Viral Hepatitis	22	10
Chen	Health service utilization of heroin abusers: A retrospective cohort study	2015	Addictive Behaviors	45	NA
Darke	Health Service Utilization among Heroin Users: 11-Year Follow-up of the Australian Treatment Outcome Study Cohort	2015	Addictive Disorders and their Treatment	14	3
Krupski	Clinical needs of patients with problem drug use	2015	Journal of the American Board of Family Medicine	28	5
Nambiar	Mortality in the Melbourne injecting drug user cohort study (MIX)	2015	Harm Reduction Journal	12	1
O'Brien	Health, perceived quality of life and health services use among homeless illicit drug users.	2015	Drug and alcohol dependence	154	NA
Onyeka	Hospitalization in a cohort seeking treatment for illicit drug use in finland	2015	Journal of Substance Abuse Treatment	53	NA
Pavarin	Health status of users of the Bologna local health authority drug addiction treatment services: a study of hospital admissions in the period 2004-2013.	2015	Le infezioni in medicina	23	1
White	Drugs-related death soon after hospital- discharge among drug treatment clients in Scotland: Record linkage, validation, and investigation of risk-factors	2015	PLoS ONE	10	11
Whittaker	Multiply disadvantaged: Health and service utilisation factors faced by homeless injecting drug consumers in Australia	2015	Drug and Alcohol Review	34	4
Bhandari	Marijuana users do not have increased healthcare utilization: A National Health and Nutrition Examination Survey (NHANES) study	2016	European Journal of Internal Medicine	34	NA
Huynh	Factors Influencing the Frequency of Emergency Department Utilization by Individuals with Substance Use Disorders	2016	Psychiatric Quarterly	87	4

First author	Title	Year	Journal	Volume	Issue
Lintzeris	Substance use, health status and service utilisation of older clients attending specialist drug and alcohol services	2016	Drug and Alcohol Review	35	2
Lo-Ciganic	Association between trajectories of buprenorphine treatment and emergency department and in-patient utilization.	2016	Addiction	111	5
Lubman	Characteristics of individuals presenting to treatment for primary alcohol problems versus other drug problems in the Australian patient pathways study	2016	BMC Psychiatry	16	1
Mohlman	Impact of Medication-Assisted Treatment for Opioid Addiction on Medicaid Expenditures and Health Services Utilization Rates in Vermont	2016	Journal of Substance Abuse Treatment	67	NA
Tran	Economic vulnerability of methadone maintenance patients: Implications for policies on co-payment services	2016	International Journal of Drug Policy	31	NA
Wilkins	An exploratory study of the health harms and utilisation of health services of frequent legal high users under the interim regulated legal high market in central Auckland	2016	New Zealand Medical Journal	129	1431
Campbell	The role of marijuana use disorder in predicting emergency department and inpatient encounters: A retrospective cohort study	2017	Drug and Alcohol Dependence	178	NA
Choi	Older adults' marijuana use, injuries, and emergency department visits	2017	American Journal of Drug and Alcohol Abuse	NA	NA
Cucciare	Longitudinal associations between outpatient medical care use and substance use among rural stimulant users	2017	American Journal of Drug and Alcohol Abuse	NA	NA
Decker	Long-term outcomes after residential substance use treatment: Relapse, morbidity, and mortality.	2017	Military Medicine	182	1
Graham	How Much Do Mental Health and Substance Use/Addiction Affect Use of General Medical Services? Extent of Use, Reason for Use, and Associated Costs	2017	Canadian Journal of Psychiatry	62	1
Kendall	A cohort study examining emergency department visits and hospital admissions among people who use drugs in Ottawa, Canada	2017	Harm Reduction Journal	14	1
Nambiar	A prospective cohort study of hospital separations among people who inject drugs in Australia: 2008-2013	2017	BMJ Open	7	8
Nambiar	Frequent emergency department presentations among people who inject drugs: A record linkage study	2017	International Journal of Drug Policy	44	NA
Nguyen	Quality of life and healthcare service utilization among methadone maintenance patients in a mountainous area of Northern Vietnam.	2017	Health and Quality of Life Outcomes	15	1
Bahorik	Alcohol, marijuana, and opioid use disorders: 5-Year patterns and characteristics of emergency department encounters.	2018	Substance abuse	0	0

First author	Title	Year	Journal	Volume	Issue
Beaulieu	Major depressive disorder and access to health services among people who use illicit drugs in Vancouver, Canada.	2018	Substance Abuse Treatment, Prevention, and Policy	0	0
Choi	Impact of depression and recreational drug use on emergency department encounters and hospital admissions among people living with HIV in Ontario: A secondary analysis using the OHTN cohort study.	2018	PLoS ONE	0	0
Han	Marijuana use by middle-aged and older adults in the United States, 2015-2016.[Erratum appears in Drug Alcohol Depend. 2018 Sep 25;192:171; PMID: 30266000]	2018	Drug & Alcohol Dependence	0	0
Manhapra	Three-year retention in buprenorphine treatment for opioid use disorder among privately insured adults.	2018	Psychiatric Services	0	0
Robertson	Associations between pharmacotherapy for opioid dependence and clinical and criminal justice outcomes among adults with co-occurring serious mental illness.	2018	Journal of Substance Abuse Treatment	0	0
Shah	Healthcare utilization and costs associated with treatment for opioid dependence.	2018	Journal of Medical Economics	0	0
Shcherbakova	Treatment Persistence Among Insured Patients Newly Starting Buprenorphine/Naloxone for Opioid Use Disorder.	2018	Annals of Pharmacotherapy	0	0

4 Stratified forest plots

ED rates by predominant drug

Study	Events	Time	Events	95%−CI	Weight
**Drugs = Injecting opiates**					
Chen (2015): Injecting opiates, China,	298.00	1578.00	18.88	[16.86; 21.16]	0.0%
Pollack (2002): Injecting opiates, United States	319.00	769.00	41.48	[37.17; 46.29]	4.0%
Nambiar (2017): Injecting opiates, Australia	2044.00	2155.00	94.85	[90.83; 99.05]	4.0%
Juday (2003): Injecting opiates, United States	4369.00	4458.00	98.00	[95.14; 100.95]	4.0%
O'Brien (2015): Injecting opiates, Ireland, Homeless	29.90	17.50	170.86	[119.39; 244.51]	0.0%
Aitken (2013): Injecting opiates, Australia	132.00	57.00	231.58	[195.26; 274.65]	4.0%
Wall (2000): Injecting opiates, Canada	323.00	114.00	283.33	[254.06; 315.98]	4.0%
**Random effects model**			**119.86**	**[82.64; 173.84]**	**19.9%**
Heterogeneity: $I^2 = 99\%$, $\tau^2 = 0.1770$, $p < 0.01$					
**Drugs = Mixed**					
Choi (2018): Mixed, Canada, HIV+	575.00	1492.70	38.52	[35.50; 41.80]	0.0%
Marshall (2012): Mixed, Canada, Young people (14−23)	229.00	427.00	53.63	[47.11; 61.05]	0.0%
French (2000): Mixed, United States	304.00	384.00	79.17	[70.75; 88.59]	4.0%
Leukefeld (2006): Mixed, United States, Male prisoners	394.00	492.00	80.08	[72.55; 88.39]	0.0%
Krupski (2015): Mixed, United States	2137.00	1696.00	126.00	[120.77; 131.46]	4.0%
Martinez (2006): Mixed, United States	1317.00	944.00	139.51	[132.18; 147.25]	4.0%
Kerr (2005): Mixed, Canada	2643.00	1766.00	149.66	[144.06; 155.48]	4.0%
Graham (2017): Mixed, Canada	19575.00	9915.00	197.43	[194.68; 200.21]	4.0%
Kendall (2017): Mixed, Canada	1404.00	663.00	211.76	[200.97; 223.14]	4.0%
Schmidt (2011): Mixed, Denmark, Schizophrenia	2577.00	369.00	698.37	[671.92; 725.86]	0.0%
**Random effects model**			**144.03**	**[117.05; 177.24]**	**24.0%**
Heterogeneity: $I^2 = 99\%$, $\tau^2 = 0.0663$, $p < 0.01$					
**Drugs = Stimulants**					
Siegal (2006): Stimulants, United States	643.00	999.00	64.36	[59.58; 69.54]	4.0%
Morasco (2014): Stimulants, United States, Veterans	4020.80	3590.00	112.00	[108.59; 115.52]	0.0%
Decker (2017): Stimulants, United States, Veterans	1325.00	1035.00	128.02	[121.31; 135.10]	0.0%
Cederbaum (2014): Stimulants, United States	12495.00	6017.00	207.66	[204.05; 211.33]	4.0%
**Random effects model**			**115.68**	**[36.71; 364.59]**	**8.0%**
Heterogeneity: $I^2 = 100\%$, $\tau^2 = 0.6852$, $p < 0.01$					
**Drugs = Injecting stimulants**					
French (2000): Injecting stimulants, United States	425.00	542.00	78.41	[71.30; 86.23]	4.0%
Reynolds (2003): Injecting stimulants, United States	516.00	600.50	85.93	[78.83; 93.67]	4.0%
Palepu (2001): Injecting stimulants, Canada	2763.00	971.75	284.33	[273.93; 295.13]	4.0%
Fairbairn (2012): Injecting stimulants, Canada, HIV+	2461.00	232.00	1060.78	[1019.68; 1103.52]	0.0%
**Random effects model**			**124.29**	**[47.96; 322.10]**	**12.0%**
Heterogeneity: $I^2 = 100\%$, $\tau^2 = 0.7066$, $p < 0.01$					
**Drugs = Opiate treatment**					
Schwarz (2012): Opiate treatment, United States	194.00	209.00	92.82	[80.64; 106.85]	4.0%
Shah (2018): Opiate treatment, United States	28219.00	29235.00	96.52	[95.41; 97.66]	4.0%
McCarty (2010): Opiate treatment, United States	8567.00	6490.00	132.00	[129.24; 134.83]	4.0%
Baser (2011): Opiate treatment, United States	14823.00	5257.00	281.97	[277.46; 286.54]	4.0%
Lo−Ciganic (2016): Opiate treatment, United States	45531.00	10945.00	416.00	[412.19; 419.84]	4.0%
Manhapra (2018): Opiate treatment, United States	95901.00	16190.00	592.35	[588.61; 596.11]	4.0%
**Random effects model**			**208.73**	**[109.73; 397.06]**	**24.0%**
Heterogeneity: $I^2 = 100\%$, $\tau^2 = 0.6449$, $p = 0$					
**Drugs = Opiates**					
Lynch (2014): Opiates, United States	7621.00	6040.00	126.18	[123.37; 129.04]	4.0%
Mohlman (2016): Opiates, United States	15052.64	8652.00	173.98	[171.22; 176.78]	4.0%
McCarty (2010): Opiates, United States	18070.00	6125.00	295.02	[290.75; 299.35]	4.0%
**Random effects model**			**186.41**	**[115.59; 300.61]**	**12.0%**
Heterogeneity: $I^2 = 100\%$, $\tau^2 = 0.1783$, $p = 0$					
**Random effects model**			**151.13**	**[113.87; 200.58]**	**100.0%**
Heterogeneity: $I^2 = 100\%$, $\tau^2 = 0.5203$, $p = 0$					

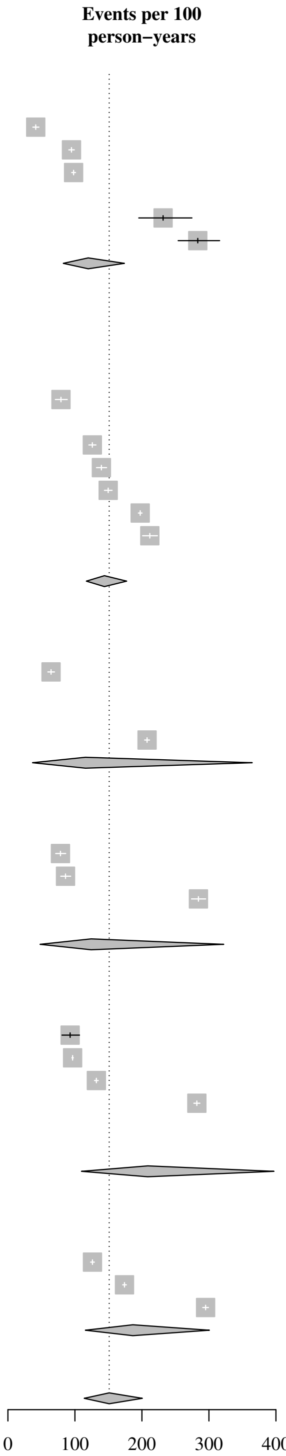

ED rates by country

Study	Events	Time	Events	95%−CI	Weight
**Country = Other**					
Chen (2015): Injecting opiates, China,	298.00	1578.00	18.88	[16.86; 21.16]	0.0%
**Random effects model**					**0.0%**
Heterogeneity: not applicable					
**Country = Canada**					
Choi (2018): Mixed, Canada, HIV+	575.00	1492.70	38.52	[35.50; 41.80]	0.0%
Marshall (2012): Mixed, Canada, Young people (14−23)	229.00	427.00	53.63	[47.11; 61.05]	0.0%
Kerr (2005): Mixed, Canada	2643.00	1766.00	149.66	[144.06; 155.48]	4.0%
Graham (2017): Mixed, Canada	19575.00	9915.00	197.43	[194.68; 200.21]	4.0%
Kendall (2017): Mixed, Canada	1404.00	663.00	211.76	[200.97; 223.14]	4.0%
Wall (2000): Injecting opiates, Canada	323.00	114.00	283.33	[254.06; 315.98]	4.0%
Palepu (2001): Injecting stimulants, Canada	2763.00	971.75	284.33	[273.93; 295.13]	4.0%
Fairbairn (2012): Injecting stimulants, Canada, HIV+	2461.00	232.00	1060.78	[1019.68; 1103.52]	0.0%
**Random effects model**			**218.45**	**[178.67; 267.07]**	**20.0%**
Heterogeneity: $I^2 = 99\%$, $\tau^2 = 0.0517$, $p < 0.01$					
**Country = United States**					
Pollack (2002): Injecting opiates, United States	319.00	769.00	41.48	[37.17; 46.29]	4.0%
Siegal (2006): Stimulants, United States	643.00	999.00	64.36	[59.58; 69.54]	4.0%
French (2000): Injecting stimulants, United States	425.00	542.00	78.41	[71.30; 86.23]	4.0%
French (2000): Mixed, United States	304.00	384.00	79.17	[70.75; 88.59]	4.0%
Leukefeld (2006): Mixed, United States, Male prisoners	394.00	492.00	80.08	[72.55; 88.39]	0.0%
Reynolds (2003): Injecting stimulants, United States	516.00	600.50	85.93	[78.83; 93.67]	4.0%
Schwarz (2012): Opiate treatment, United States	194.00	209.00	92.82	[80.64; 106.85]	4.0%
Shah (2018): Opiate treatment, United States	28219.00	29235.00	96.52	[95.41; 97.66]	4.0%
Juday (2003): Injecting opiates, United States	4369.00	4458.00	98.00	[95.14; 100.95]	4.0%
Morasco (2014): Stimulants, United States, Veterans	4020.80	3590.00	112.00	[108.59; 115.52]	0.0%
Krupski (2015): Mixed, United States	2137.00	1696.00	126.00	[120.77; 131.46]	4.0%
Lynch (2014): Opiates, United States	7621.00	6040.00	126.18	[123.37; 129.04]	4.0%
Decker (2017): Stimulants, United States, Veterans	1325.00	1035.00	128.02	[121.31; 135.10]	0.0%
McCarty (2010): Opiate treatment, United States	8567.00	6490.00	132.00	[129.24; 134.83]	4.0%
Martinez (2006): Mixed, United States	1317.00	944.00	139.51	[132.18; 147.25]	4.0%
Mohlman (2016): Opiates, United States	15052.64	8652.00	173.98	[171.22; 176.78]	4.0%
Cederbaum (2014): Stimulants, United States	12495.00	6017.00	207.66	[204.05; 211.33]	4.0%
Baser (2011): Opiate treatment, United States	14823.00	5257.00	281.97	[277.46; 286.54]	4.0%
McCarty (2010): Opiates, United States	18070.00	6125.00	295.02	[290.75; 299.35]	4.0%
Lo−Ciganic (2016): Opiate treatment, United States	45531.00	10945.00	416.00	[412.19; 419.84]	4.0%
Manhapra (2018): Opiate treatment, United States	95901.00	16190.00	592.35	[588.61; 596.11]	4.0%
**Random effects model**			**136.66**	**[96.54; 193.44]**	**72.0%**
Heterogeneity: $I^2 = 100\%$, $\tau^2 = 0.5647$, $p = 0$					
**Country = Australia**					
Nambiar (2017): Injecting opiates, Australia	2044.00	2155.00	94.85	[90.83; 99.05]	4.0%
Aitken (2013): Injecting opiates, Australia	132.00	57.00	231.58	[195.26; 274.65]	4.0%
**Random effects model**			**147.62**	**[61.55; 354.02]**	**8.0%**
Heterogeneity: $I^2 = 99\%$, $\tau^2 = 0.3944$, $p < 0.01$					
**Country = Europe**					
O'Brien (2015): Injecting opiates, Ireland, Homeless	29.90	17.50	170.86	[119.39; 244.51]	0.0%
Schmidt (2011): Mixed, Denmark, Schizophrenia	2577.00	369.00	698.37	[671.92; 725.86]	0.0%
**Random effects model**					**0.0%**
Heterogeneity: not applicable					
**Random effects model**			**151.13**	**[113.87; 200.58]**	**100.0%**
Heterogeneity: $I^2 = 100\%$, $\tau^2 = 0.5203$, $p = 0$					

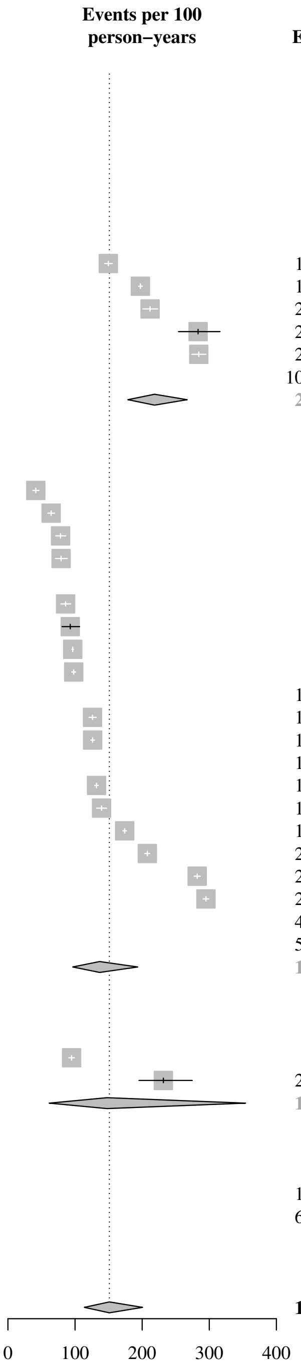

Hospital admission rates by predominant drug

Study	Events	Time	Events	95%−CI	Weight
**Drugs = Injecting opiates**					
Chen (2015): Injecting opiates, China,	138.00	1578.00	8.75	[7.40; 10.33]	0.0%
Lloyd−Smith (2010): Injecting opiates, Canada	442.00	1931.00	22.89	[20.85; 25.13]	4.0%
Juday (2003): Injecting opiates, United States	4355.00	10888.00	40.00	[38.83; 41.20]	4.0%
Wall (2000): Injecting opiates, Canada	120.00	114.00	105.26	[88.02; 125.89]	4.0%
**Random effects model**			**45.52**	**[26.03; 79.61]**	**12.0%**
Heterogeneity: $I^2 = 99\%$, $\tau^2 = 0.2405$, $p < 0.01$					
**Drugs = Mixed**					
Choi (2018): Mixed, Canada, HIV+	130.00	1492.70	8.71	[7.33; 10.34]	0.0%
Kendall (2017): Mixed, Canada	141.00	663.00	21.27	[18.03; 25.08]	4.0%
Leukefeld (2006): Mixed, United States, Male prisoners	118.00	492.00	23.98	[20.02; 28.73]	0.0%
White (2015): Mixed, United Kingdom	173107.00	705538.00	24.54	[24.42; 24.65]	4.0%
Martinez (2006): Mixed, United States	247.00	944.00	26.17	[23.10; 29.64]	4.0%
French (2000): Mixed, United States	119.00	384.00	30.99	[25.89; 37.09]	4.0%
Graham (2017): Mixed, Canada	3967.00	9915.00	40.01	[38.78; 41.27]	4.0%
Onyeka (2015): Mixed, Finland	22453.00	41567.50	54.02	[53.31; 54.73]	4.0%
Schmidt (2011): Mixed, Denmark, Schizophrenia	3145.00	369.00	852.30	[823.03; 882.62]	0.0%
**Random effects model**			**31.22**	**[20.50; 47.54]**	**24.0%**
Heterogeneity: $I^2 = 100\%$, $\tau^2 = 0.2729$, $p = 0$					
**Drugs = Opiate treatment**					
Schwarz (2012): Opiate treatment, United States	40.00	209.00	19.14	[14.04; 26.09]	3.9%
Schoenbaum (2002): Opiate treatment, United States	492.00	2456.40	20.03	[18.34; 21.88]	4.0%
Pavarin (2015): Opiate treatment, Italy	504.00	2171.00	23.22	[21.27; 25.33]	4.0%
McCarty (2010): Opiate treatment, United States	1558.00	6490.00	24.01	[22.84; 25.23]	4.0%
Ngo (2008): Opiate treatment, Australia	940.00	2926.00	32.13	[30.14; 34.25]	4.0%
Pavarin (2015): Opiate treatment, Italy	628.00	1760.00	35.68	[33.00; 38.58]	4.0%
Shah (2018): Opiate treatment, United States	12113.00	29235.00	41.43	[40.70; 42.18]	4.0%
Walley (2012): Opiate treatment, United States	80.00	138.00	57.97	[46.56; 72.17]	3.9%
Schoenbaum (2002): Opiate treatment, United States, HIV+	662.00	1101.70	60.09	[55.68; 64.85]	0.0%
Gourevitch (2007): Opiate treatment, United States	800.00	1161.00	68.91	[64.29; 73.85]	4.0%
Baser (2011): Opiate treatment, United States	8410.00	5257.00	159.98	[156.59; 163.43]	4.0%
Lo−Ciganic (2016): Opiate treatment, United States	17867.00	10945.00	163.24	[160.87; 165.65]	4.0%
**Random effects model**			**43.62**	**[26.22; 72.56]**	**44.0%**
Heterogeneity: $I^2 = 100\%$, $\tau^2 = 0.7378$, $p = 0$					
**Drugs = Injecting stimulants**					
French (2000): Injecting stimulants, United States	174.00	542.00	32.10	[27.67; 37.25]	4.0%
Nambiar (2017): Injecting stimulants, Australia	2106.00	4163.50	50.58	[48.47; 52.79]	4.0%
Palepu (2001): Injecting stimulants, Canada	495.00	971.75	50.94	[46.64; 55.63]	4.0%
**Random effects model**			**44.16**	**[36.04; 54.11]**	**12.0%**
Heterogeneity: $I^2 = 94\%$, $\tau^2 = 0.0296$, $p < 0.01$					
**Drugs = Opiates**					
Mohlman (2016): Opiates, United States	3144.28	8652.00	36.34	[35.09; 37.63]	4.0%
McCarty (2010): Opiates, United States	4478.00	6125.00	73.11	[71.00; 75.28]	4.0%
**Random effects model**			**51.55**	**[25.99; 102.26]**	**8.0%**
Heterogeneity: $I^2 = 100\%$, $\tau^2 = 0.2440$, $p < 0.01$					
**Random effects model**			**41.02**	**[29.77; 56.51]**	**100.0%**
Heterogeneity: $I^2 = 100\%$, $\tau^2 = 0.6648$, $p = 0$					

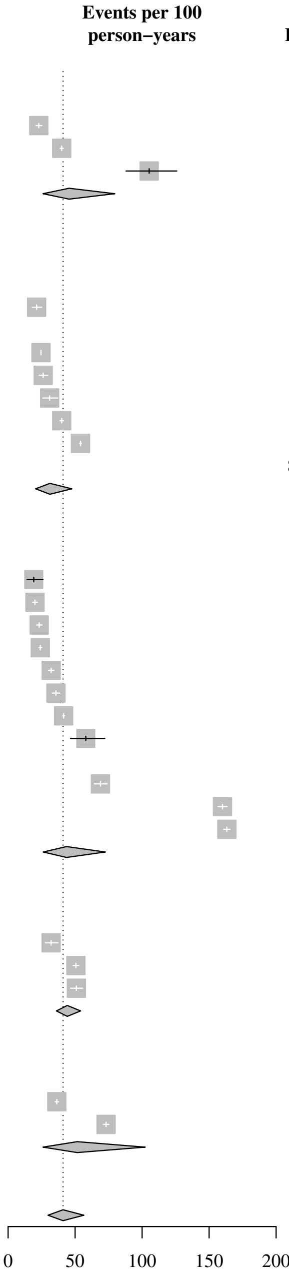

Hospital admission rates by country

Study	Events	Time	Events	95%−CI	Weight
**country2 = Other**					
Chen (2015): Injecting opiates, China,	138.00	1578.00	8.75	[7.40; 10.33]	0.0%
**Random effects model**					**0.0%**
Heterogeneity: not applicable					
**country2 = Canada**					
Choi (2018): Mixed, Canada, HIV+	130.00	1492.70	8.71	[7.33; 10.34]	0.0%
Kendall (2017): Mixed, Canada	141.00	663.00	21.27	[18.03; 25.08]	4.0%
Lloyd−Smith (2010): Injecting opiates, Canada	442.00	1931.00	22.89	[20.85; 25.13]	4.0%
Graham (2017): Mixed, Canada	3967.00	9915.00	40.01	[38.78; 41.27]	4.0%
Palepu (2001): Injecting stimulants, Canada	495.00	971.75	50.94	[46.64; 55.63]	4.0%
Wall (2000): Injecting opiates, Canada	120.00	114.00	105.26	[88.02; 125.89]	4.0%
**Random effects model**			**40.03**	**[27.99; 57.24]**	**20.0%**
Heterogeneity: $I^2 = 99\%$, $\tau^2 = 0.1626$, $p < 0.01$					
**country2 = United States**					
Schwarz (2012): Opiate treatment, United States	40.00	209.00	19.14	[14.04; 26.09]	3.9%
Schoenbaum (2002): Opiate treatment, United States	492.00	2456.40	20.03	[18.34; 21.88]	4.0%
Leukefeld (2006): Mixed, United States, Male prisoners	118.00	492.00	23.98	[20.02; 28.73]	0.0%
McCarty (2010): Opiate treatment, United States	1558.00	6490.00	24.01	[22.84; 25.23]	4.0%
Martinez (2006): Mixed, United States	247.00	944.00	26.17	[23.10; 29.64]	4.0%
French (2000): Mixed, United States	119.00	384.00	30.99	[25.89; 37.09]	4.0%
French (2000): Injecting stimulants, United States	174.00	542.00	32.10	[27.67; 37.25]	4.0%
Mohlman (2016): Opiates, United States	3144.28	8652.00	36.34	[35.09; 37.63]	4.0%
Juday (2003): Injecting opiates, United States	4355.00	10888.00	40.00	[38.83; 41.20]	4.0%
Shah (2018): Opiate treatment, United States	12113.00	29235.00	41.43	[40.70; 42.18]	4.0%
Walley (2012): Opiate treatment, United States	80.00	138.00	57.97	[46.56; 72.17]	3.9%
Schoenbaum (2002): Opiate treatment, United States, HIV+	662.00	1101.70	60.09	[55.68; 64.85]	0.0%
Gourevitch (2007): Opiate treatment, United States	800.00	1161.00	68.91	[64.29; 73.85]	4.0%
McCarty (2010): Opiates, United States	4478.00	6125.00	73.11	[71.00; 75.28]	4.0%
Baser (2011): Opiate treatment, United States	8410.00	5257.00	159.98	[156.59; 163.43]	4.0%
Lo−Ciganic (2016): Opiate treatment, United States	17867.00	10945.00	163.24	[160.87; 165.65]	4.0%
**Random effects model**			**44.37**	**[29.41; 66.94]**	**55.9%**
Heterogeneity: $I^2 = 100\%$, $\tau^2 = 0.6119$, $p = 0$					
**country2 = Europe**					
Pavarin (2015): Opiate treatment, Italy	504.00	2171.00	23.22	[21.27; 25.33]	4.0%
White (2015): Mixed, United Kingdom	173107.00	705538.00	24.54	[24.42; 24.65]	4.0%
Pavarin (2015): Opiate treatment, Italy	628.00	1760.00	35.68	[33.00; 38.58]	4.0%
Onyeka (2015): Mixed, Finland	22453.00	41567.50	54.02	[53.31; 54.73]	4.0%
Schmidt (2011): Mixed, Denmark, Schizophrenia	3145.00	369.00	852.30	[823.03; 882.62]	0.0%
**Random effects model**			**32.38**	**[18.96; 55.32]**	**16.1%**
Heterogeneity: $I^2 = 100\%$, $\tau^2 = 0.2977$, $p = 0$					
**country2 = Australia**					
Ngo (2008): Opiate treatment, Australia	940.00	2926.00	32.13	[30.14; 34.25]	4.0%
Nambiar (2017): Injecting stimulants, Australia	2106.00	4163.50	50.58	[48.47; 52.79]	4.0%
**Random effects model**			**40.34**	**[25.85; 62.94]**	**8.0%**
Heterogeneity: $I^2 = 99\%$, $\tau^2 = 0.1023$, $p < 0.01$					
**Random effects model**			**41.02**	**[29.77; 56.51]**	**100.0%**
Heterogeneity: $I^2 = 100\%$, $\tau^2 = 0.6648$, $p = 0$					

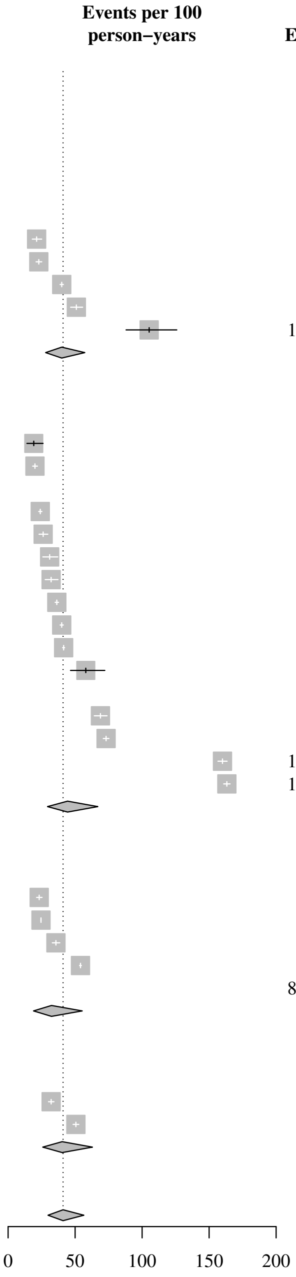

5 PRISMA checklist

Section	#	Checklist item	Reported on page
TITLE			
Title	1	Identify the report as a systematic review, meta-analysis, or both.	p1
ABSTRACT			
Structured summary	2	Provide a structured summary including, as applicable: background; objectives; data sources; study eligibility criteria, participants, and interventions; study appraisal and synthesis methods; results; limitations; conclusions and implications of key findings; systematic review registration number.	p2
INTRODUCTION			
Rationale	3	Describe the rationale for the review in the context of what is already known.	p3
Objectives	4	Provide an explicit statement of questions being addressed with reference to participants, interventions, comparisons, outcomes, and study design (PICOS).	p4
METHODS			
Protocol and registration	5	Indicate if a review protocol exists, if and where it can be accessed (e.g., Web address), and, if available, provide registration information including registration number.	p4
Eligibility criteria	6	Specify study characteristics (e.g., PICOS, length of follow-up) and report characteristics (e.g., years considered, language, publication status) used as criteria for eligibility, giving rationale.	p4 (no intervention)
Information sources	7	Describe all information sources (e.g., databases with dates of coverage, contact with study authors to identify additional studies) in the search and date last searched.	p4
Search	8	Present full electronic search strategy for at least one database, including any limits used, such that it could be repeated.	Suppl. Info.
Study selection	9	State the process for selecting studies (i.e., screening, eligibility, included in systematic review, and, if applicable, included in the meta-analysis).	p4
Data collection process	10	Describe method of data extraction from reports (e.g., piloted forms, independently, in duplicate) and any processes for obtaining and confirming data from investigators.	p4
Data items	11	List and define all variables for which data were sought (e.g., PICOS, funding sources) and any assumptions and simplifications made.	p4
Risk of bias in individual studies	12	Describe methods used for assessing risk of bias of individual studies (including specification of whether this was done at the study or outcome level), and how this information is to be used in any data synthesis.	p4
Summary measures	13	State the principal summary measures (e.g., risk ratio, difference in means).	p4
Synthesis of results	14	Describe the methods of handling data and combining results of studies, if done, including measures of consistency (e.g., I2) for each meta-analysis.	p5

Section	#	Checklist item	Reported on page
Risk of bias across studies	15	Specify any assessment of risk of bias that may affect the cumulative evidence (e.g., publication bias, selective reporting within studies).	p4
Additional analyses	16	Describe methods of additional analyses (e.g., sensitivity or subgroup analyses, meta-regression), if done, indicating which were pre-specified.	p5
RESULTS			
Study selection	17	Give numbers of studies screened, assessed for eligibility, and included in the review, with reasons for exclusions at each stage, ideally with a flow diagram.	p6
			Fig. 1
Study characteristics	18	For each study, present characteristics for which data were extracted (e.g., study size, PICOS, follow-up period) and provide the citations.	Suppl. Info.
Risk of bias within studies	19	Present data on risk of bias of each study and, if available, any outcome level assessment (see item 12).	Table 1 Suppl. Info.
Results of individual studies	20	For all outcomes considered (benefits or harms), present, for each study: (a) simple summary data for each intervention group (b) effect estimates and confidence intervals, ideally with a forest plot.	pp7-8
			Figs. 4-5
Synthesis of results	21	Present results of each meta-analysis done, including confidence intervals and measures of consistency.	p8
Risk of bias across studies	22	Present results of any assessment of risk of bias across studies (see Item 15).	p6
			Table 1
Additional analysis	23	Give results of additional analyses, if done (e.g., sensitivity or subgroup analyses, meta-regression [see Item 16]).	pp7-9
DISCUSSION			
Summary of evidence	24	Summarize the main findings including the strength of evidence for each main outcome; consider their relevance to key groups (e.g., healthcare providers, users, and policy makers).	p9
Limitations	25	Discuss limitations at study and outcome level (e.g., risk of bias), and at review-level (e.g., incomplete retrieval of identified research, reporting bias).	p10
Conclusions	26	Provide a general interpretation of the results in the context of other evidence, and implications for future research.	pp10-11
FUNDING			
Funding	27	Describe sources of funding for the systematic review and other support (e.g., supply of data); role of funders for the systematic review.	p1
